# Responses to docetaxel plus vinorelbine in metastatic breast cancer patients failing high-dose chemotherapy

**DOI:** 10.1038/sj.bjc.6600164

**Published:** 2002-03-18

**Authors:** R Andres, J I Mayordomo, D Isla, J L Marti, P Escudero, E Filipovich, A Saenz, I Alvarez, E Polo, A Tres

**Affiliations:** División of Medical Oncology, Hospital Clinco Universitario, Av. San Juan Bosco, 15, E-50009, Zaragoza, Spain

## Abstract

*British Journal of Cancer* (2002) **86**, 1017–1018. DOI: 10.1038/sj/bjc/6600164 www.bjcancer.com

© 2002 Cancer Research UK

## Sir

A number of breast cancer patients have been treated worldwide with high-dose chemotherapy (HDCT) with stem cell support worldwide. The survival advantage of this aggressive approach is controversial.

The treatment of breast cancer patients with relapse following high-dose chemotherapy is a major challenge for the oncologist. Response rates have been negligible with regimens containing anthracyclines, alkylating agents and antimetabolites. We have tested a combination of docetaxel plus virorelbine, two drugs that are not commonly included in the induction or consolidation phases of high-dose chemotherapy programs.

From January 1997 to April 2001, 30 consenting patients relapsing after HDCT have been treated with docetaxel 60 mg m^2^ i.v. in 1 h plus vinorelbine 24 mg m^2^, both given on day 1 of a 21-day course, with standard premedication. Patients were monitored for neurotoxicity every three courses with full neurological examination, electroneurography, electromiography and somatosensorial evoked potentials. Median age was 48 years (range 33–66). All patients had been previously treated with anthracyclines. Prior treatment included HDCT with stem cell rescue in all patients (given as adjuvant treatment in six patients and for metastases in 22).

When given as adjuvant treatment for high-risk stage II–III breast cancer (six patients), HDCT consisted of cyclophosphamide, tiothepa, and carboplatin (CTCb) as previously described (Antman *et al*, 1992). An antracycline-containing regimen (fluorouracil, epirubicin and cyclophosphamide) (FEC-75) had been given as induction prior to HDCT. In 24 patients treated with HDCT for metastases, CTCb was combined with high-dose paclitaxel (Mayordomo *et al*, 1997; Iñiguez *et al*, 1998; Isla *et al*, 1998). Tamoxifen had been given, starting right after HDCT, to 22 patients (73%). Median disease-free interval from HDCT was 12 months (range: 3–47): 17 months for adjuvant HDCT and 12 for those treated for metastases. Sites of disease upon protocol entry included bone in 15 patients, liver in 13, lung in eight, lymph nodes in six, skin and soft tissue in four and bone marrow in one. Eighteen patients had metastases to one organ, six to two and six to three or more. Median performance status was one (range 0–2). All patients were evaluable for response, measured with standard criteria (Miller *et al*, 1981). There were four complete responses (13.3%) and 14 partial responses (46.6%) for a response rate of 60%. Five patients (16.6%) had stable disease and seven (23.3%) progressive disease. Response rates were not different for patients pretreated with HDCT as adjuvant versus those treated for metastases and for those receiving HDCT without paclitaxel. With median follow-up of 30 months or to death, time to progression in all patients is 7 months (range 1–40+). Median survival is 12 months (range 1–40+). Seven patients are currently alive. Toxicity was manageable. In 137 courses delivered (median six per patient, range 1 to 9), there were the following grade 3–4 toxicities: alopecia (137 courses, 100%), neutropenia (nine courses, 6.5%), febrile neutropenia (eight courses, 5.8%) and anemia (two courses, 1.4%). No cases of grade 3–4 neurotoxicity were seen.

High-dose chemotherapy with alkylating agents and stem-cell support is the most active treatment in patients with metastatic breast cancer. However there is controversy on whether HDCT has a survival advantage over conventional chemotherapy (Lotz *et al*, 1999; Stadtmauer *et al*, 1999). One of the reasons for this discrepancy may be the lack of active chemotherapy regimens after progression to HDCT.

Most patients undergoing aggressive HDCT programs receive an induction with conventional-dose anthracyclin containing chemotherapy, followed by HDCT with two or three alkylating agents. So it is not surprising that conventional chemotherapy with anthracyclines or alkylators, including cisplatin for relapsed metastatic breast cancer after HDCT yields poor response rates.

The emergence of novel active agents in breast cancer such as taxanes and vinorelbine has changed the scenario for these patients (in addition to challenging the whole concept of HDCT, unless these agents can be incorporated into it and response rates and survival with higher doses are proven to be superior to conventional doses). Sola *et al* reported 68% responses with paclitaxel in 28 relapsed patients (Sola *et al*, 1999). Sundaran *et al* reported five responses in seven evaluable patients with capecitabine (Sundaran *et al*, 2000).

Docetaxel plus vinorelbeine is a promising combination in this heavily pretreated population with 60% responses, even in patients pretreated with paclitaxel. Toxicity was surprisingly acceptable and neurotoxicity was not a major clinical problem. The short duration of responses is the major challenge. No doubt, more active novel drugs are needed to improve or sustain these responses, especially in patients with shorter disease-free interval.
